# Gender Differences in Patients with Atrial Fibrillation Receiving Oral Anticoagulants

**DOI:** 10.31083/j.rcm2503092

**Published:** 2024-03-06

**Authors:** Jo-Nan Liao, Yu-Shan Huang, Chuan-Tsai Tsai, Ling Kuo, Su-Jung Chen, Ta-Chuan Tuan, Tzeng-Ji Chen, Shih-Ann Chen, Tze-Fan Chao

**Affiliations:** ^1^Division of Cardiology, Department of Medicine, Taipei Veterans General Hospital, 11217 Taipei, Taiwan; ^2^Institute of Clinical Medicine, and Cardiovascular Research Center, National Yang Ming Chiao Tung University, 30010 Taipei, Taiwan; ^3^Women’s Heart Section, Cardiovascular Center, Taipei Veterans General Hospital, 11217 Taipei, Taiwan; ^4^Institute of Public Health and School of Medicine, National Yang Ming Chiao Tung University, 30010 Taipei, Taiwan.; ^5^Division of Infectious Diseases, Department of Medicine, Taipei Veterans General Hospital, 11217 Taipei, Taiwan; ^6^Department of Family Medicine, Taipei Veterans General Hospital Hsinchu Branch, 31064 Zhudong, Taiwan; ^7^Cardiovascular Center, Taichung Veterans General Hospital, 40705 Taichung, Taiwan

**Keywords:** atrial fibrillation, NOAC, gender difference

## Abstract

**Background:**

Gender is a well-recognized risk factor in atrial fibrillation (AF)-related ischemic stroke. The association of gender with the use of oral anticoagulants (OACs) and prognosis remains unknown.

**Methods:**

The National Health Insurance Research Database in Taiwan identified 203,775 patients with AF aged ≥20 years from 2012 to 2018, with 55.4% of males. Our main study cohort included 67,426 patients using OACs. The study endpoints include death, ischemic stroke, intracranial hemorrhage, major bleeding, and composite adverse events.

**Results:**

Significant differences were found in baseline characteristics between sexes. Female patients with AF were older and had higher ${\mathrm{CHA}}_{2}$${\mathrm{DS}}_{2}$-VASc and HAS-BLED scores. Non-vitamin K antagonist oral anticoagulant (NOAC) use was more prominent in females while the use of warfarin was similar in both sexes. The distribution of baseline characteristics between the warfarin and NOAC groups in both sexes was much alike. Among the whole study cohort, NOAC was associated with a decreased risk of clinical endpoints compared to warfarin, which remained the same in subgroup analyses of both sexes. Additionally, a greater risk reduction of ischemic stroke with NOAC was observed in female patients compared to male patients (adjusted hazard ratio: 0.517 in males, 0.425 in females, interaction *p* = 0.040).

**Conclusions:**

This nationwide cohort demonstrated the differences between male and female patients with AF, including baseline characteristics, risk profiles, and medication use. Despite great differences in baseline demographic data, NOAC was associated with better clinical outcomes compared to warfarin in both sexes, and females benefited more than males in preventing ischemic stroke using NOACs.

## 1. Introduction

Atrial fibrillation (AF) is the most prevalent sustained cardiac arrhythmia, and it increases risks of ischemic stroke, intracranial hemorrhage (ICH), and mortality [1, 2, 3]. Sex differences in terms of clinical presentations, risk profile, response to treatment, and prognosis are observed in patients with AF [4]. In particular, women with AF frequently experience more symptomatic AF episodes, have worse quality of life, more drug-related arrhythmias, and are less likely to take oral anticoagulants (OACs) [5, 6, 7, 8, 9]. Among patients undergoing catheter ablation for AF, female sex was independently associated with a higher risk of adverse events [10] and more frequent AF recurrences [11]. Further, a higher mortality rate is observed in female patients with AF. Furthermore, the female sex is considered as a disease modifier for AF-related ischemic stroke and contributes one point in the ${\mathrm{CHA}}_{2}$${\mathrm{DS}}_{2}$-VASc score to guide the use of OAC [12]. Therefore, the 2020 European Society of Cardiology (ESC) guidelines for AF have a distinct section addressing sex-related differences in AF which underscoring the significance of recognizing and resolving sex-specific barriers to implementing guideline-recommended treatments for AF [12]. Moreover, the guidelines recommend that women and men with AF are equally offered therapies to prevent stroke [12]. Currently, non-vitamin K antagonist oral anticoagulant (NOAC) is recommended for preventing AF-related ischemic stroke [12, 13, 14] because of its superior safety and comparable or even better efficacy in randomized controlled trials (RCTs) and real-world cohort studies [15, 16, 17]. Our previous study demonstrated a gradual increase of OAC prescription after the introduction of NOAC in 2012: from 13.6% in 2008 Q1 to 35.6% in 2015 Q3. Warfarin use decreased from 13.6% to 9.6%, whereas NOAC use increased from 0% to 26% from 2008 to 2015 [18]. However, whether NOAC-related risk reduction differs between sexes remains unknown. Therefore, we aim to use a nationwide AF cohort to investigate gender differences in terms of OAC types and related prognosis.

## 2. Methods

### 2.1 Database

The present study used the “National Health Insurance Research Database (NHIRD)” released by the Taiwan National Health Research Institutes. The National Health Insurance (NHI) system is a mandatory universal health insurance program that provides comprehensive medical care coverage to all Taiwanese residents. The NHIRD consists of detailed healthcare data from >23 million enrollees, representing >99% of Taiwan’s population. The cohort dataset has encrypted the patients’ original identification numbers to protect their privacy, and the encrypting procedure was consistent so that a linkage of the claims belonging to the same patient was feasible within the NHI database and could be continuously followed.

### 2.2 Study Cohort

**Fig. 1.**
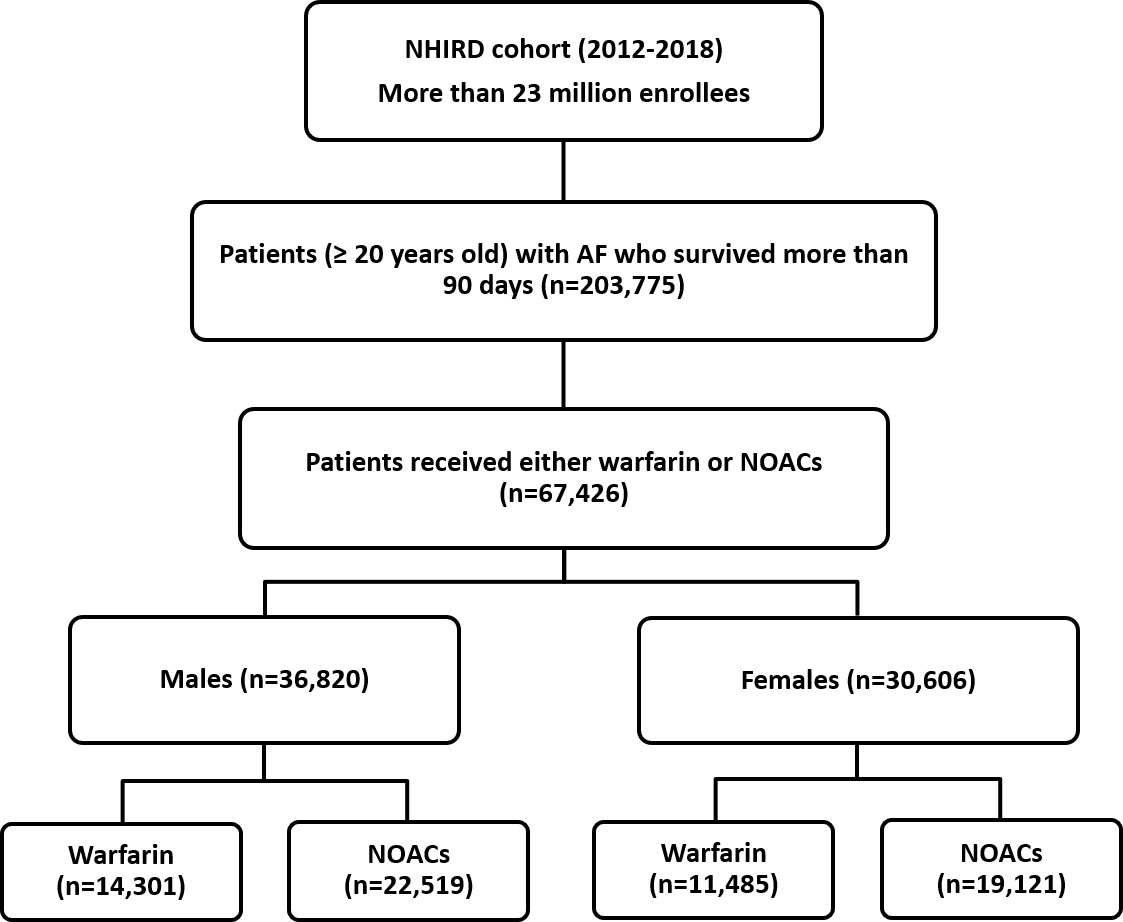
**Patient enrollment flowchart**. A total of 203,775 patients aged ≥20 years with AF were identified from the nationwide cohort and 67,426 of them were taking either warfarin or NOACs. Further comparisons between males and females in terms of types of oral anticoagulants were performed. AF, atrial fibrillation; NHIRD, National Health Insurance Research Database; NOACs, non-vitamin K antagonist oral anticoagulants.

The study protocol is similar to our previous studies which have been published [19, 20]. Patients aged ≥20 years with AF identified from the NHIRD from 2012 to 2018 constituted the main study population. Fig. 1 shows the flowchart of the patient enrollment and study design.

The International Classification of Diseases, Ninth Revision, Clinical Modification (ICD-9-CM) codes were used to confirm the diagnosis. We defined patients with a certain disease only when it was a discharge diagnosis or confirmed more than twice in the outpatient department to ensure the accuracy of diagnosis [19, 20, 21]. The ${\mathrm{CHA}}_{2}$${\mathrm{DS}}_{2}$-VASc score was calculated for each patient by assigning 1 point each for ages 65–74 years and a history of hypertension (HTN), diabetes mellitus (DM), heart failure (HF), vascular disease (myocardial infarction or peripheral artery disease), and female gender and 2 points each for a history of a stroke, transient ischemic attack (TIA), and age of ≥75 years [22]. The HAS-BLED score was calculated by assigning 1 point each for hypertension, abnormal renal, or liver function, stroke, bleeding history, age ≥65 years, and antiplatelet drug or alcohol use [23]. The information on the international normalized ratio (INR) of warfarin was unavailable in the Taiwan registry database, so the scoring in the present study excluded the component of “labile INR”, consistent with prior registry studies. Additionally, abnormal renal or liver function was defined by the ICD-9-CM codes rather than laboratory data. The diagnostic accuracy using this definition in NHIRD has been validated previously [24, 25, 26].

### 2.3 Clinical Endpoints

The clinical endpoints included the occurrence of death, ischemic stroke, intracerebral hemorrhage (ICH), major bleeding, and composite adverse events (death or ischemic stroke or ICH or major bleeding). Ischemic stroke and ICH were diagnosed with concomitant brain imaging studies, including computed tomography or magnetic resonance imaging. Major bleeding was ICH or bleeding originating from the gastrointestinal, genitourinary, or respiratory tract that requires hospitalization. Each endpoint was independently analyzed of the others without being censored. The accuracy of diagnosis of ischemic stroke in Taiwan’s NHIRD was approximately 94% [27]. Another validation study demonstrated that the diagnostic accuracy of ischemic stroke in NHIRD was high, with a positive predictive value and sensitivity of 88.4% and 97.3%, respectively [28].

### 2.4 Statistical Analysis

Data were presented as the mean value and standard deviation for normally distributed continuous variables and proportions for categorical variables. The unpaired two-tailed *t*-test was used to assess differences between continuous values. Nominal variables were compared by chi-square test or Fisher’s exact test. Incidence rates of events were calculated by dividing the number of events by person-year at risk. The Kaplan-Meier method was used to plot the cumulative incidence of clinical events with statistical significance examined by the log-rank test. Multivariate Cox proportional hazards models were used for risk prediction adjusting for significant baseline variables. All statistical significances were set at a *p*-value of <0.05 using the Statistical Package for the Social Sciences version 17.0 statistical software (SPSS, Chicago, IL, USA).

## 3. Results

### 3.1 Baseline Characteristics

Our study population consisted of 203,775 patients aged ≥20 years with AF from 2012 to 2018 and 112,836 (55.4%) of them were males. Compared to males, females were older, had more comorbidities of HF, HTN, DM, previous stroke/TIA, hyperlipidemia, autoimmune diseases, anemia, history of bleeding and less vascular disease, chronic obstructive pulmonary disease, cancer, abnormal liver function, and alcohol excess/abuse (Table 1). Therefore, females demonstrated higher ${\mathrm{CHA}}_{2}$${\mathrm{DS}}_{2}$-VASc and HAS-BLED scores. The ${\mathrm{CHA}}_{2}$${\mathrm{DS}}_{2}$-VASc scores remained higher in females after excluding the one point contributed by the female gender. Males were more likely to use anti-platelet drugs while slightly but significantly more females were taking NOACs, but warfarin use demonstrated no difference between sexes. Males were apt to use beta-blockers for rate control while females tended to take calcium channel blockers and digoxin. A higher percentage of dronedarone and propafenone use was found in females regarding rhythm control agents (Table 1).

**Table 1.** **Baseline characteristics of male and female AF patients**.

Variables		All	Males	Females	*p* value
		(n = 203,775)	(n =112,836)	(n = 90,939)	
Age, years; mean value (SD)		72.76 (13.52)	70.7 (13.94)	75.3 (12.53)	<0.0001
Age ≥75 years, n (%)		102,641 (50.37)	49,041 (43.46)	53,600 (58.94)	<0.0001
Age 65–74 years, n (%)		48,290 (23.7)	27,961 (24.78)	20,329 (22.35)	<0.0001
${\mathrm{CHADS}}_{2}$ score		2.49 (1.56)	2.33 (1.54)	2.69 (1.56)	<0.0001
${\mathrm{CHA}}_{2}$${\mathrm{DS}}_{2}$-VASc score		3.8 (2.00	3.15 (1.87)	4.62 (1.84)	<0.0001
${\mathrm{CHA}}_{2}$${\mathrm{DS}}_{2}$-VASc score (no gender)		3.36 (1.87)	3.15 (1.87)	3.62 (1.84)	<0.0001
HAS-BLED score		2.91 (1.43)	2.85 (1.47)	2.98 (1.37)	<0.0001
Comorbidities, n (%)					
	Congestive heart failure	73,989 (36.31)	37,600 (33.32)	36,389 (40.01)	<0.0001
	Hypertension	158,518 (77.79)	84,836 (75.19)	73,682 (81.02)	<0.0001
	Diabetes mellitus	73,302 (35.97)	38,349 (33.99)	34,953 (38.44)	<0.0001
	Previous stroke/TIA	49,939 (24.51)	26,779 (23.73)	23,160 (25.47)	<0.0001
	Vascular diseases	24,759 (12.15)	14,596 (12.94)	10,163 (11.18)	<0.0001
	COPD	54,270 (26.63)	34,114 (30.23)	20,156 (22.16)	<0.0001
	Hyperlipidemia	94,780 (46.51)	49,339 (43.73)	45,441 (49.97)	<0.0001
	Autoimmune diseases	13,017 (6.39)	4831 (4.28)	8186 (9)	<0.0001
	Cancer	26,249 (12.88)	15,366 (13.62)	10,883 (11.97)	<0.0001
	Abnormal renal function	42,276 (20.75)	23,574 (20.89)	18,702 (20.57)	0.0703
	Abnormal liver function	41,364 (20.3)	23,964 (21.24)	17,400 (19.13)	<0.0001
	Anemia	31,954 (15.68)	14,207 (12.59)	17,747 (19.52)	<0.0001
	History of bleeding	58,866 (28.89)	32,331 (28.65)	26,535 (29.18)	0.0093
	Alcohol excess/abuse, n (%)	3677 (1.8)	3271 (2.9)	406 (0.45)	<0.0001
Use of NSAIDs, n (%)		9316 (4.57)	5109 (4.53)	4207 (4.63)	0.2911
Use of anti-platelet drugs, n (%)		83,559 (41.01)	49,168 (43.57)	34,391 (37.82)	<0.0001
Aspirin		64,326 (31.57)	38,792 (34.38)	25,534 (28.08)	<0.0001
Clopidogrel		19,463 (9.55)	11,834 (10.49)	7629 (8.39)	<0.0001
Dipyridamole		7830 (3.84)	4250 (3.77)	3580 (3.94)	0.0475
Ticlopidine		3368 (1.65)	1746 (1.55)	1622 (1.78)	<0.0001
Anticoagulant					
	Warfarin	27,971 (13.73)	15,504 (13.74)	12,467 (13.71)	0.8392
	NOACs	43,825 (21.51)	23,722 (21.02)	20,103 (22.11)	<0.0001
Rate-control agents					
	Beta-blockers	88,936 (43.64)	47,562 (42.15)	12,014 (13.21)	<0.0001
	CCBs	25,487 (12.51)	13,473 (11.94)	12,017 (13.21)	<0.0001
	Digoxin	25,801 (12.66)	13,784 (12.22)	12,014 (13.21)	<0.0001
Rhythm-control agents					
	Amiodarone	42,467 (20.84)	23,543 (20.86)	18,924 (20.81)	0.7602
	Dronedarone	4469 (2.19)	2123 (1.88)	2346 (2.58)	<0.0001
	Propafenone	18,088 (8.88)	9757 (8.65)	8331 (9.16)	<0.0001
	Flecainide	900 (0.44)	501 (0.44)	399 (0.44)	0.8589
	Sotalol	368 (0.18)	207 (0.18)	161 (0.18)	0.7343
	ACEIs/ARBs	88,257 (43.31)	47,935 (42.48)	40,322 (44.34)	<0.0001
	Statins	38,455 (18.87)	21,131 (18.73)	17,324 (19.05)	0.0642

ACEIs/ARBs, angiotensin converting enzyme inhibitors/angiotensin receptor blockers; CCBs, calcium channel blockers; COPD, chronic obstructive pulmonary disease; NOACs, non-vitamin K antagonist oral anticoagulants; AF, atrial fibrillation; NSAIDs, non-steroidal anti-inflammatory drugs; SD, standard deviation; TIA, transient ischemic attack.

### 3.2 Gender Differences in Baseline Characteristics in Terms of Warfarin or NOAC Use

Among all patients with AF, 36,820 males and 30,606 females taking OACs were further analyzed (Table 2). The distribution of baseline characteristics between warfarin and NOAC users was very similar between the sexes. Both male and female patients taking NOAC were older and had more underlying comorbidities except HF, abnormal renal function, anemia, and a history of bleeding compared to those using warfarin. NOAC users demonstrated higher ${\mathrm{CHA}}_{2}$${\mathrm{DS}}_{2}$-VASc and HAS-BLED scores. The ${\mathrm{CHA}}_{2}$${\mathrm{DS}}_{2}$-VASc scores remained higher in NOAC users than in warfarin users in both sexes even after excluding the point contributed by gender. Anti-platelet drug use was more common in warfarin users in both sexes, but clopidogrel use was more common in male NOAC users and female warfarin users. Female NOAC users were more likely to take beta-blockers than female warfarin users, whereas the percentage of beta-blockers used was similar between NOAC and warfarin groups in males.

**Table 2.** **Baseline characteristics between warfarin and NOAC users in male and female patients with AF**.

Variables		All (n = 67,426)		*p* value	Males (n = 36,820)		*p* value	Females (n = 30,606)		*p* value
		Warfarin	NOACs		Warfarin	NOACs		Warfarin	NOACs	
		(n = 25,786)	(n = 41,640)		(n =14,301)	(n = 22,519)		(n = 11,485)	(n = 19,121)	
Age, years; mean value (SD)		70.7 (13.94)	75.86 (10.65)	<0.0001	68.22 (12.92)	74.00 (11.24)	<0.0001	72.35 (12.15)	78.05 (9.46)	<0.0001
Age ≥75 years, n (%)		10,332 (40.07)	24,673 (59.25)	<0.0001	4832 (33.79)	11,632 (51.65)	<0.0001	5500 (47.89)	13,041 (68.2)	<0.0001
Age 65–74 years, n (%)		6821 (26.45)	11,449 (27.5)	0.003	3837 (26.83)	6788 (30.14)	<0.0001	2984 (25.98)	4661 (24.38)	0.0018
Sex (male), n (%)		14,301 (55.46)	22,519 (54.08)	0.0005	-	-	-	-	-	-
${\mathrm{CHADS}}_{2}$ score		2.33 (1.54)	2.76 (1.43)	<0.0001	2.34 (1.50)	2.62 (1.42)	<0.0001	2.61 (1.56)	2.93 (1.42)	<0.0001
${\mathrm{CHA}}_{2}$${\mathrm{DS}}_{2}$-VASc score		3.15 (1.87)	4.21 (1.75)	<0.0001	3.06 (1.83)	3.56 (1.63)	<0.0001	4.45 (1.85)	4.96 (1.57)	<0.0001
${\mathrm{CHA}}_{2}$${\mathrm{DS}}_{2}$-VASc score (no gender)		3.15 (1.87)	3.75 (1.62)	<0.0001	3.06 (1.83)	3.56 (1.63)	<0.0001	3.45 (1.85)	3.96 (1.57)	<0.0001
HAS-BLED score		2.85 (1.47)	2.89 (1.27)	<0.0001	2.58 (1.46)	2.86 (1.33)	<0.0001	2.66 (1.39)	2.93 (1.19)	<0.0001
Comorbidities, n (%)										
	Congestive heart failure	11,309 (43.86)	15,705 (37.72)	<0.0001	5865 (41.01)	7942 (35.27)	<0.0001	5444 (47.4)	7763 (40.6)	<0.0001
	Hypertension	19,359 (75.08)	34,814 (83.61)	<0.0001	10,596 (74.09)	18,256 (81.07)	<0.0001	8763 (76.3)	16,558 (86.6)	<0.0001
	Diabetes mellitus	8827 (34.23)	16,003 (38.43)	<0.0001	4728 (33.06)	8307 (36.89)	<0.0001	4099 (35.69)	7696 (40.25)	<0.0001
	Previous stroke/TIA	6844 (26.54)	11,906 (28.59)	<0.0001	3743 (26.17)	6415 (28.49)	<0.0001	3101 (27)	5491 (28.72)	0.0011
	Vascular diseases	2758 (10.7)	4884 (11.73)	<0.0001	1648 (11.52)	2856 (12.68)	0.0008	1110 (9.66)	2028 (10.61)	0.0079
	COPD	5791 (22.46)	10,693 (25.68)	<0.0001	3565 (24.93)	6647 (29.52)	<0.0001	2226 (19.38)	4046 (21.16)	0.0002
	Hyperlipidemia	11,761 (45.61)	22,495 (54.02)	<0.0001	6221 (43.5)	11,562 (51.34)	<0.0001	5540 (48.24)	10,933 (57.18)	<0.0001
	Autoimmune diseases	1492 (5.79)	2833 (6.8)	<0.0001	528 (3.69)	1027 (4.56)	<0.0001	964 (8.39)	1806 (9.45)	0.0017
	Cancer	2635 (10.22)	5387 (12.94)	<0.0001	1494 (10.45)	3131 (13.9)	<0.0001	1141 (9.93)	2256 (11.8)	<0.0001
	Abnormal renal function	5237 (20.31)	7543 (18.11)	<0.0001	2971 (20.77)	4312 (19.15)	0.0001	2266 (19.73)	3231 (16.9)	<0.0001
	Abnormal liver function	4785 (18.56)	8743 (21)	<0.0001	2807 (19.63)	4850 (21.54)	<0.0001	1978 (17.22)	3893 (20.36)	<0.0001
	Anemia	3692 (14.32)	4825 (11.59)	<0.0001	1584 (11.08)	2172 (9.65)	<0.0001	2108 (18.35)	2653 (13.87)	<0.0001
	History of bleeding	6713 (26.03)	11,353 (27.26)	0.0004	3583 (25.05)	6285 (27.91)	<0.0001	3130 (27.25)	5068 (26.5)	0.1534
	Alcohol excess/abuse, n (%)	414 (1.61)	557 (1.34)	0.0055	378 (2.64)	491 (2.18)	0.0052	36 (0.31)	66 (0.35)	0.6371
Use of NSAIDs, n (%)		1047 (4.06)	1647 (3.96)	0.4999	603 (4.22)	878 (3.9)	0.1339	444 (3.87)	769 (4.02)	0.4966
Use of anti-platelet drugs, n (%)		6480 (25.13)	7719 (18.54)	<0.0001	4003 (27.99)	4733 (21.02)	<0.0001	2477 (21.57)	2986 (15.62)	<0.0001
Aspirin		4726 (18.33)	5045 (12.12)	<0.0001	2999 (20.97)	3094 (13.74)	<0.0001	1727 (15.04)	1951 (10.2)	<0.0001
Clopidogrel		1521 (5.9)	2462 (5.91)	0.9401	957 (6.69)	1629 (7.23)	0.0455	564 (4.91)	833 (4.36)	0.0266
Dipyridamole		795 (3.08)	977 (2.35)	<0.0001	437 (3.06)	567 (2.52)	0.0025	358 (3.12)	410 (2.14)	<0.0001
Ticlopidine		285 (1.11)	193 (0.46)	<0.0001	166 (1.16)	111 (0.49)	<0.0001	119 (1.04)	82 (0.43)	<0.0001
Rate-control agents										
	Beta-blockers	13,041 (50.57)	21,695 (52.1)	0.0001	7106 (49.69)	11,268 (50.04)	0.514	5935 (51.68)	10,427 (54.53)	<0.0001
	CCBs	3255 (12.62)	5688 (13.66)	0.0001	1708 (11.94)	2846 (12.64)	0.0471	1547 (13.47)	2842 (14.86)	0.0007
	Digoxin	5721 (22.19)	4828 (11.59)	<0.0001	2995 (20.94)	2519 (11.19)	<0.0001	2726 (23.74)	2309 (12.08)	<0.0001
Rhythm-control agents										
	Amiodarone	7054 (27.36)	8970 (21.54)	<0.0001	3879 (27.12)	4726 (20.99)	<0.0001	3175 (27.64)	4244 (22.2)	<0.0001
	Dronedarone	529 (2.05)	1458 (3.5)	<0.0001	248 (1.73)	704 (3.13)	<0.0001	281 (2.45)	754 (3.94)	<0.0001
	Propafenone	1909 (7.4)	3959 (9.51)	<0.0001	1080 (7.55)	1945 (8.64)	0.0002	829 (7.22)	2014 (10.53)	<0.0001
	Flecainide	94 (0.36)	294 (0.71)	<0.0001	64 (0.45)	155 (0.69)	0.0021	30 (0.26)	139 (0.73)	<0.0001
	Sotalol	105 (0.41)	89 (0.21)	<0.0001	63 (0.44)	51 (0.23)	0.0008	42 (0.37)	38 (0.2)	0.0101
	ACEIs/ARBs	12,161 (47.16)	22,666 (54.43)	<0.0001	6896 (48.22)	12,029 (53.42)	<0.0001	5265 (45.84)	10,637 (55.63)	<0.0001
	Statins	5136 (19.92)	10,775 (25.88)	<0.0001	2809 (19.64)	5963 (26.48)	<0.0001	2327 (20.26)	4812 (25.17)	<0.0001

ACEIs/ARBs, angiotensin converting enzyme inhibitors/angiotensin receptor blockers; CCBs, calcium channel blockers; COPD, chronic obstructive pulmonary disease; NOACs, non-vitamin K antagonist oral anticoagulants; AF, atrial fibrillation; NSAIDs, non-steroidal anti-inflammatory drugs; SD, standard deviation; TIA, transient ischemic attack.

### 3.3 Clinical Endpoints in Terms of OAC Types and Sexes

**Fig. 2.**
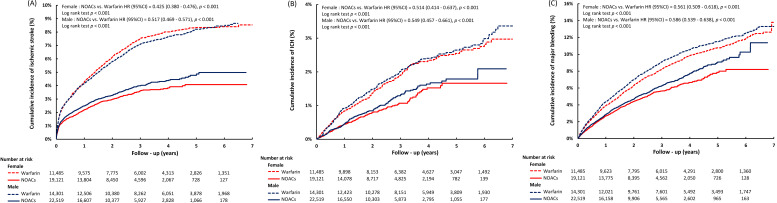
**Cumulative incidence curves of ischemic stroke (A), ICH (B), and major bleeding (C) in male and female patients in relation to OAC use**. The c Kaplan-Meier analysis revealed higher cumulative incidence rates in the NOAC group compared to the warfarin group for both sexes. Additionally, the reduction in the cumulative incidence of ischemic stroke with NOAC use, as opposed to warfarin, was more pronounced in female patients than in male patients. ICH, intracranial hemorrhage; NOACs, non-vitamin K antagonist oral anticoagulants; OAC, oral anticoagulants; CI, confidence interval; HR, hazard ratio.

The mean follow-up of 2.89 years reported 12,850 deaths, 3033 ischemic strokes, 874 ICHs, 4125 major bleeding, and 16,750 composite adverse events. Compared to warfarin use, the NOAC group had lower incidence rates of death (6.99% versus 7.32%), ischemic stroke (1.47% versus 2.07%), ICH (0.40% versus 0.58%), major bleeding (2.17% versus 2.65%), and composite adverse events (9.80% versus 10.65%). The Kaplan-Meier analysis demonstrated higher rates of clinical events in the NOAC group compared to the warfarin group for both sexes, with female patients exhibiting a more prominent decrease in cumulative incidence of ischemic stroke with NOAC use compared to warfarin (Fig. 2).

**Fig. 3.**
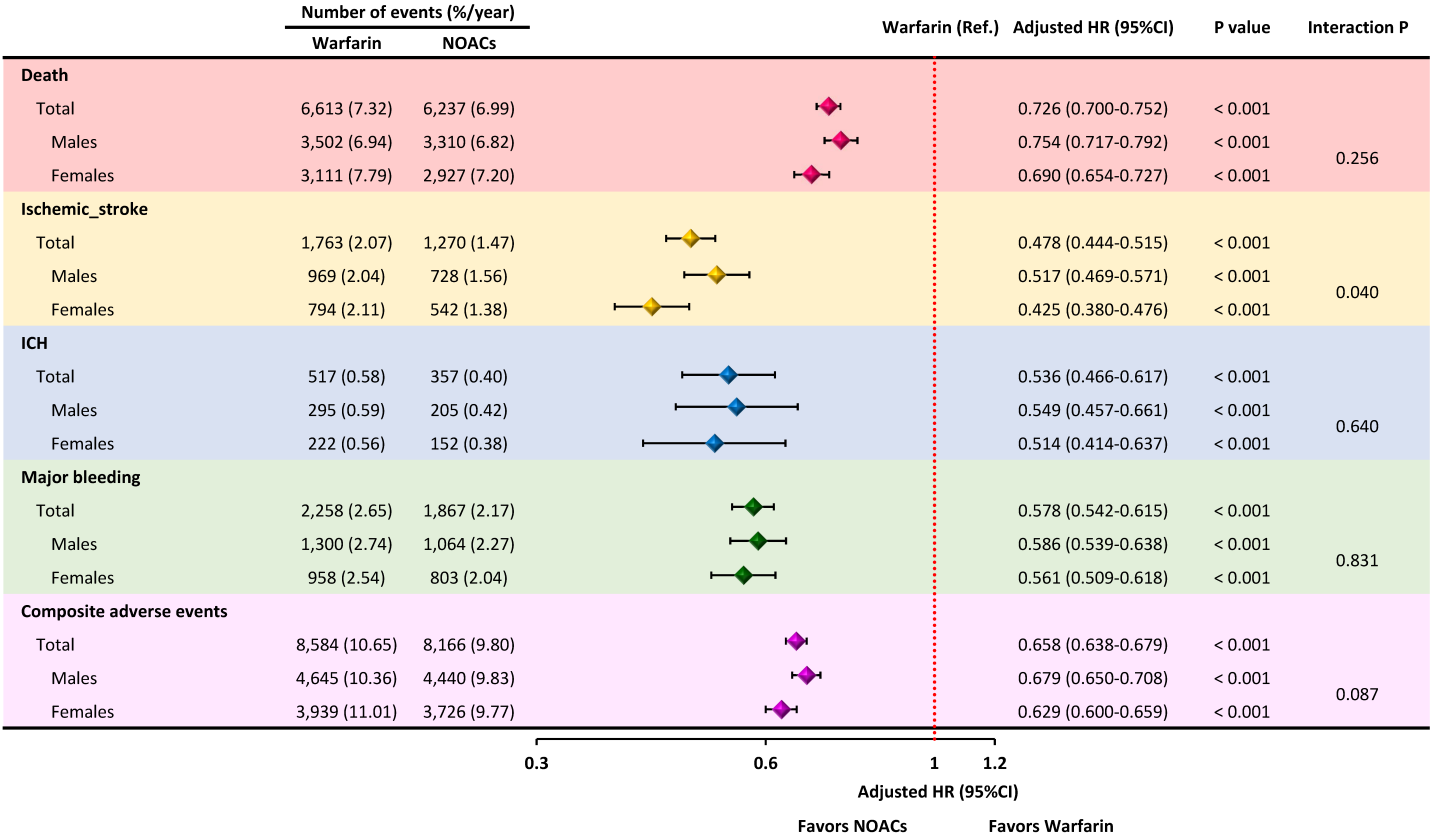
**Incidence and risk of clinical endpoints between warfarin and NOAC use in both males and females**. The whole study cohort demonstrated that NOAC was associated with lower risks of clinical endpoints compared to warfarin. Subgroup analysis revealed the consistently better outcomes associated with NOAC compared to warfarin in both sexes, whereas a greater risk reduction of ischemic stroke was observed in female patients with AF. AF, atrial fibrillation; CI, confidence interval; HR, hazard ratio; ICH, intracranial hemorrhage; NOACs, non-vitamin K antagonist oral anticoagulants.

Multivariate Cox regression analysis revealed that NOAC was associated with lower risk of death (adjusted hazard rate [aHR]: 0.726, 95% confidence interval [CI]: 0.700–0.752, *p* < 0.001), ischemic stroke (aHR: 0.478, 95% CI: 0.444–0.515, *p* < 0.001), ICH (aHR: 0.536, 95% CI: 0.466–0.617, *p* < 0.001), major bleeding (aHR: 0.578, 95% CI: 0.542–0.615, *p* < 0.001), and composite adverse events (aHR: 0.658, 95% CI: 0.628–0.679, *p* < 0.001) (Fig. 3). Subgroup analyses between males and females were performed in terms of OAC types. NOACs demonstrated a consistent association with lower risks of death, ischemic stroke, ICH, major bleeding, and composite adverse events than warfarin in both sexes. Moreover, risk reduction of ischemic stroke with NOAC compared to warfarin was significantly greater in females than in males (interaction *p* = 0.040) (Fig. 3).

## 4. Discussion

### 4.1 Main Findings

This nationwide cohort analyzed the characteristics and long-term prognosis of male and female patients with AF in terms of OAC types and presented the following main results: (1) gender differences in baseline characteristics and medication use in patients with AF, where female patients with AF demonstrated higher ${\mathrm{CHA}}_{2}$${\mathrm{DS}}_{2}$-VASc and HAS-BLED scores; (2) the distribution of underlying demographic characteristics between warfarin and NOAC groups was much similar between male and female patients with AF; (3) the observation that NOAC was associated with better outcomes compared to warfarin was consistent in both sexes, and female patients with AF demonstrated a greater risk reduction of ischemic stroke.

### 4.2 OAC Use Differed between Sexes

Data in terms of OAC use in both sexes differ in previous reports. The CARMEN-AF registry [29] and the Global Anticoagulant Registry in the Field (GARFIELD-AF) [30] revealed no gender differences in OAC use. Conversely, the United States PINNACLE National Cardiovascular Data Registry from 2008 to 2014 reported that women with AF were more likely to receive aspirin but not OACs [31]. The present study revealed slight but significant differences in OAC prescription because NOAC use was more common in females and the percentage of warfarin use was similar between sexes. We hypothesized that a constellation of multiple factors, such as different periods, geographic factors, and underlying demographics caused gender differences in OAC use. For example, vascular diseases were more common in males and thus more males received aspirin or clopidogrel than women did. The need for multiple blood thinners might be a crucial factor for doctors while selecting medications.

### 4.3 Gender Differences in NOAC-Associated Risk Reduction

Generally, female patients with AF have a higher risk of stroke and systemic thromboembolism, and AF-related embolic stroke in women is more severe and disabling [32, 33, 34]. Warfarin was the mainstream of stroke prevention in patients with AF before the introduction of NOAC, but the Medicare administrative claims data revealed that warfarin reduced stroke less well in females. Further, female patients with AF had a slightly higher risk of hospitalizations despite warfarin use [35]. One possible explanation underlying this observation is the higher chance of poor INR control in females [36]. Until now, no RCTs have compared gender differences with OAC use. The DIRECT registry, a single-center prospective observational registry of 806 patients with AF treated with NOACs, demonstrated comparable bleeding events between men and women whereas the thromboembolic event rate was higher in women [37]. One meta-analysis, including major RCTs of NOACs versus warfarin in patients with AF (RE-LY, ROCKET-AF, ARISTOTLE, and AVERROES), revealed a higher risk of systemic thromboembolism in females compared to males when treated with warfarin, which did not occur with NOAC treatment [38]. One review article revealed that the sex disparity in stroke is no longer seen after introducing NOAC [38, 39]. Furthermore, one meta-analysis reported differential benefits of NOACs between sexes in which male patients were more protected from stroke/systemic thromboembolism and female patients from major bleeding events [40]. Our present study was partly congruent with previous studies that both sexes benefited from NOAC despite different background characteristics, and the unfavorable prognosis in females no longer existed with NOAC. However, we revealed a greater risk reduction of ischemic stroke with NOAC use in female patients despite higher ${\mathrm{CHA}}_{2}$${\mathrm{DS}}_{2}$-VASc scores. Potential reasons underlying the different beneficial effects of NOAC between sexes in previous studies are unknown, probably due to the different study designs and cohorts, age distributions, and background characteristics. In particular, the meta-analysis included some trials that were not powerful enough to evaluate sex-specific differences. An RCT specifically designed for the evaluation of gender differences in terms of NOAC use is required for a robust conclusion.

### 4.4 Study Limitations

There are some limitations in the present study. First, males and females might possess different biochemistry data and demographic information, which were lacking in the database, but this was a common limitation in the registry database. Second, the diagnosis and occurrence of events were based on the diagnostic codes registered by the physicians responsible for patient treatments, and under-diagnosis could be excluded. However, the accuracy of diagnosis in Taiwan’s NHIRD has been previously validated [24, 25, 27, 28]. Third, INR levels and time in the therapeutic range of warfarin use were not available in the database. Fourth, because this is a retrospective observational study, the reasons underlying more risk reduction of ischemic stroke with NOAC in female patients is unknown. We postulated the benefit of NOAC over warfarin might be more prominent in females because female patients with AF were more likely to have poor INR control than male patients in previous study [25]. However, this is solely an assumption because INR data is not available in the present study. Finally, the doses and types of NOACs were not analyzed in our study, thus whether or not these factors would interfere with the results remains unknown.

## 5. Conclusions

This large-scale nationwide cohort revealed that the use of NOAC was associated with better long-term outcomes compared with warfarin in patients with AF in both sexes. Female patients with AF benefited more from NOAC in reducing ischemic stroke, regardless of a higher risk. More studies are required for solid results about gender differences in the era of NOAC and for possible mechanisms.

## Data Availability

All data generated or analyzed during this study are included in this published article.
